# A Phytoremediation Efficiency Assessment of Cadmium (Cd)-Contaminated Soils in the Three Gorges Reservoir Area, China

**DOI:** 10.3390/plants14142202

**Published:** 2025-07-16

**Authors:** Yinhua Guo, Wei Liu, Lixiong Zeng, Liwen Qiu, Di Wu, Hao Wen, Rui Yuan, Dingjun Zhang, Rongbin Tang, Zhan Chen

**Affiliations:** 1 China Three Gorges Corporation National Engineering Research Center of Eco-Environment in the Yangtze River Economic Belt, Wuhan 430010, China; guo_yinhua@126.com (Y.G.); wu_di3@ctg.com.cn (D.W.); yuan_rui3@ctg.com.cn (R.Y.); zhang_dingjun@ctg.com.cn (D.Z.); tang_rongbin@ctg.com.cn (R.T.); 2 Hubei Key Laboratory of Rare Resource Plants in Three Gorges Reservoir Area, Yichang 443000, China; 3 Key Laboratory of Forest Ecology and Environment of National Forestry and Grassland Administration, Ecology and Nature Conservation Institute, Chinese Academy of Forestry, Beijing 100091, China; liuwei_10@yeah.net (W.L.); shuzijunjerry@163.com (H.W.); chenzhan0508@caf.ac.cn (Z.C.); 4 Hubei Zigui Three Gorges Reservoir National Forest Ecosystem Observation and Research Station, Zigui 443600, China; 5 College of Horticulture and Gardening, Yangtze University, Jingzhou 434000, China

**Keywords:** cadmium contamination, Three Gorges Reservoir Area, phytoremediation, remediation efficacy

## Abstract

To investigate the remediation efficiency of different plant species on cadmium (Cd)-contaminated soil, this study conducted a pot experiment with two woody species (*Populu adenopoda* and *Salix babylonica*) and two herbaceous species (*Artemisia argyi* and *Amaranthus hypochondriacus*). Soils were collected from an abandoned coal mine and adjacent pristine natural areas within the dam-adjacent section of the Three Gorges Reservoir Area to establish three soil treatment groups: unpolluted soil (T1, 0.18 mg·kg^−1^ Cd), a 1:1 mixture of contaminated and unpolluted soil (T2, 0.35 mg·kg^−1^ Cd), and contaminated coal mine soil (T3, 0.54 mg·kg^−1^ Cd). This study aimed to investigate the growth status of plants, Cd accumulation and translocation characteristics, and the relationship between them and soil environmental factors. Woody plants exhibited significant advantages in aboveground biomass accumulation. Under T3 treatment, the Cd extraction amount of *S. babylonica* (224.93 mg) increased by about 36 times compared to T1, and the extraction efficiency (6.42%) was significantly higher than other species. Among the herbaceous species, *A. argyi* showed the maximum Cd extraction amount (66.26 mg) and extraction efficiency (3.11%) during T2 treatment. While *A. hypochondriacus* exhibited a trend of increasing extraction amount but decreasing extraction efficiency with increasing concentration. With the exception of *S. babylonica* under T1 treatment (*BCF* = 0.78), the bioconcentration factor was greater than 1 in both woody (*BCF* = 1.39–6.42) and herbaceous species (*BCF* = 1.39–3.11). However, herbaceous plants demonstrated significantly higher translocation factors (*TF* = 1.58–3.43) compared to woody species (*TF* = 0.31–0.87). There was a significant negative correlation between aboveground phosphorus (P) content and root Cd (*p* < 0.05), while underground nitrogen (N) content was positively correlated to aboveground Cd content (*p* < 0.05). Soil total N and available P were significantly positively correlated with plant Cd absorption, whereas total potassium (K) showed a negative correlation. This study demonstrated that woody plants can achieve long-term remediation through biomass advantages, while herbaceous plants, with their high transfer efficiency, are suitable for short-term rotation. In the future, it is suggested to conduct a mixed planting model of woody and herbaceous plants to remediate Cd-contaminated soils in the tailing areas of reservoir areas. This would synergistically leverage the dual advantages of root retention and aboveground removal, enhancing remediation efficiency. Concurrent optimization of soil nutrient management would further improve the Cd remediation efficiency of plants.

## 1. Introduction

As the world’s largest hydro-junction project, the Three Gorges Reservoir Region constitutes as a critical water conservation area and ecological barrier in China. It plays a pivotal role in promoting the sustainable development of the Yangtze River Economic Belt [1,2]. However, rapid economic growth, accelerated urbanization, and industrialization in the region have led to increasingly severe environmental security challenges, among which cadmium (Cd) contamination has emerged as a critical concern [3,4]. Soil Cd concentrations along certain tributaries of the Three Gorges Reservoir Region have exceeded China’s risk screening threshold for soil contamination in agricultural land (0.3 mg·kg^−1^), with localized areas exhibiting an exceedance rate of 12.7% [5]. Critically, the unique topographical and hydrological characteristics of the Reservoir Region, particularly the 30 m vertical amplitude of the water level fluctuation zone (WLFZ) subjected to cyclic “winter inundation and summer exposure” regimes, significantly exacerbate the risk of Cd remobilization [6,7]. Seasonal drying–rewetting cycles induce redox fluctuations, triggering iron reduction and dissolution of Fe/Mn oxides, thereby releasing bound Cd into more bioavailable forms [8,9]. Previous studies have confirmed that Cd in WLFZ soils is characterized by low residual fractions but high labile fractions, with effective Cd content reaching up to 38.51%, presenting high environmental risks [10,11]. As a highly toxic, mobile, and bio-accumulative heavy metal, Cd can accumulate through the food chain, posing significant threats to ecosystem integrity and resident safety within the Reservoir Region [12,13]. The synergistic effects of hydrological perturbations, soil heterogeneity, and anthropogenic inputs collectively create a “chemical time bomb” that poses elevated ecological risks in the WLFZ [14,15]. Therefore, it is urgent to develop effective soil remediation technologies to mitigate Cd contamination for safeguarding regional ecological stability and public health. The unique topography and hydrology of the Three Gorges Reservoir Region exacerbate the complexity of Cd contamination. Although conventional physicochemical remediation techniques (e.g., soil replacement and chemical leaching) can rapidly reduce Cd concentrations, their high implementation costs and detrimental impacts on soil microecology render them unsuitable for addressing large-scale, low-concentration pollution scenarios characteristic of this area [16]. Consequently, developing environmentally sustainable remediation strategies has become essential for ensuring the ecological security of the Reservoir Region.

Phytoremediation, a technique utilizing hyperaccumulator plants to absorb, immobilize, or transform contaminants, demonstrates significant advantages including low operational costs, minimal environmental disturbance and biorecovery of precious and semiprecious metals [17,18,19]. However, the sustainable management of contaminated plant biomass following harvest remains a critical challenge, particularly for herbaceous species which often produce significant volumes of residue [20]. Current research is focused on exploring safe and effective disposal or valorization strategies for these metal-laden residues, including composting with stabilization agents, controlled thermal conversion (e.g., pyrolysis, gasification) to concentrate metals in ash or produce biochar, and specialized anaerobic digestion processes [21,22]. Addressing this end-of-life management is essential to prevent secondary contamination and to maximize the environmental benefits of phytoremediation. Previous research has identified multiple Cd-hyperaccumulating plant species globally, such as *Thlaspi arvense*, *Solanum nigrum*, *Lonicera japonica*, *Sedum alfredii*, and *Phytolacca acinose* [23,24,25,26,27]. However, traditional hyperaccumulators often face limitations in large-scale applications due to low biomass and poor adaptability to complex environments [28]. Plants selected for engineering applications typically exhibit enhanced traits including high accumulation capacity, elevated biomass, rapid growth, cultivation ease, and environmental resilience [29]. Studies indicate that *S. babylonica* demonstrates rapid growth, extensive root systems, strong adaptability, and high biomass, facilitating efficient heavy metal uptake and stabilization [30,31]. *P. adenopoda*, native to China’s reservoir regions, shows exceptional adaptation to Three Gorges Reservoir Region with high stress resistance, ecological stability, substantial biomass, and rapid growth [32]. Herbaceous species like *A. hypochondriacus* and *A. argyi* exhibit remarkable nutrient-poor soil tolerance, stress resistance, and simplified cultivation requirements [33,34,35,36]. Based on these considerations, this study selected these four species to evaluate their phytoremediation efficiency across Cd contamination gradients. This study aimed to elucidate the interrelationships between plant growth responses, Cd accumulation patterns, and soil physicochemical properties. The findings establish scientific foundations for developing reservoir-specific phytoremediation strategies and also provide technical references for enhancing remediation efficacy through soil nutrient management.

## 2. Results

### 2.1. Growth Responses of Woody and Herbaceous Plants

Woody plants exhibited significant increases in shoot biomass under Cd treatments (Table 1, *p* < 0.05). Notably, *P. adenopoda* showed 2.26-fold higher shoot biomass at the T3 exposure level than T1. No other physiological parameters showed statistically significant changes (*p* > 0.05). Among herbaceous species, *A. hypochondriacus* displayed significantly enhanced net growth in plant height and basal diameter under the T3 treatment relative to T1 (*p* < 0.05), whereas there were no significant differences in either shoot or root biomass (*p* > 0.05). For *A. argyi*, T3 treatment induced a marked increase in basal diameter compared to T1 (*p* < 0.05). However, plant height, shoot biomass, and root biomass showed no significant inter-treatment differences (*p* > 0.05).

### 2.2. BCF and TF

The *BCF* values for both woody and herbaceous plants exceeded 1 across all three Cd treatments, except for *S. babylonica* under T1 (Table 2). Among woody species, *S. babylonica* had a significantly lower *BCF* in T1 compared to other plants (*p* < 0.05). Notably, its *BCF* increased markedly with rising Cd concentrations (*p* < 0.05), reaching significantly higher values in T3 than the other three species. Conversely, *P. adenopoda* showed no significant differences in *BCF* across treatments (*p* > 0.05).

For herbaceous species, *A. hypochondriacus* displayed a significantly higher BCF in T1 than in T2 and T3 (*p* < 0.05). Similarly, *A. argyi* demonstrated higher BCF values in T1 and T2 compared to T3 (*p* < 0.05), though no inter-treatment differences were observed in other metrics.

### 2.3. Cd Extraction and Phytoextraction Efficiency

Cd uptake and extraction efficiency across the four plant species revealed that Cd uptake in both woody species increased significantly with rising Cd treatment levels (Table 3, *p* < 0.05). Notably, *S. babylonica* under T3 exhibited markedly higher Cd uptake than the other three species (*p* < 0.05). Conversely, no significant differences in Cd uptake were observed between treatments for the two herbaceous species (*p* > 0.05).

Regarding extraction efficiency, *P. adenopoda* showed no significant variations across Cd treatment levels (*p* > 0.05). Conversely, *S. babylonica* exhibited a concentration-dependent enhancement in Cd extraction efficiency, with values increasing significantly at higher Cd exposures (*p* < 0.05). Among herbaceous plants, *A. hypochondriacus* achieved significantly higher extraction efficiency in T1 than in T2 and T3 (*p* < 0.05). Similarly, *A. argyi* displayed greater efficiency for T1 and T2 compared to T3 (*p* < 0.05). No significant differences in extraction efficiency were observed between woody plants and herbaceous plants under T1 and T2 treatments (*p* > 0.05), but *S. babylonica* in T3 treatment displayed significantly higher extraction efficiency than the other three species (*p* < 0.05).

### 2.4. Correlation Between Plant Nutrient Uptake and Cd Content

**Figure 1:**
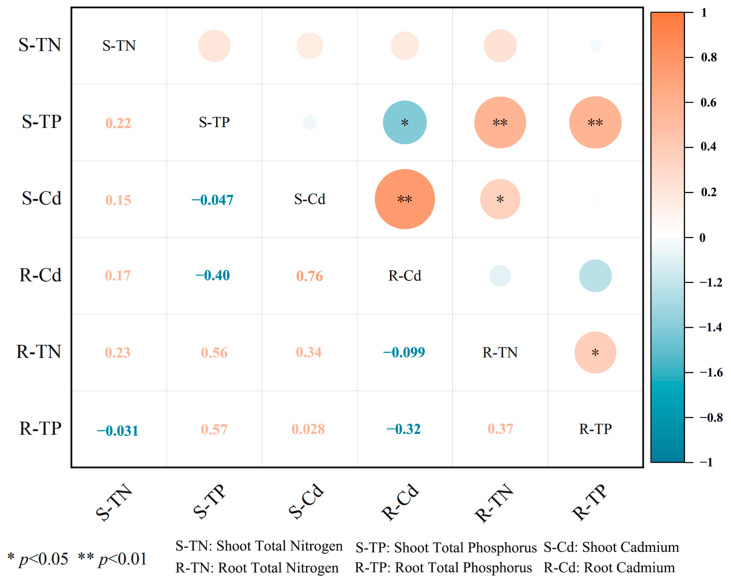
Correlation analysis between plant nutrient uptake and plant Cd extraction.

Statistical analysis of the correlation demonstrated that shoot TP (S-TP) exhibited a significantly negative correlation with root Cd (R-Cd) (*p* < 0.05), while S-TP showed extremely significant positive correlations with root TP (R-TP) and root TN (R-TN) (Figure 1, *p* < 0.01). A significantly positive correlation was observed between R-TN and shoot Cd (S-Cd) (*p* < 0.05), while R-TN was also positively correlated with R-TP (*p* < 0.05). Additionally, S-Cd and R-Cd demonstrated a strong positive correlation (*p* < 0.01).

### 2.5. Correlation Between Plant Cd Extraction and Soil Physicochemical Properties

**Figure 2:**
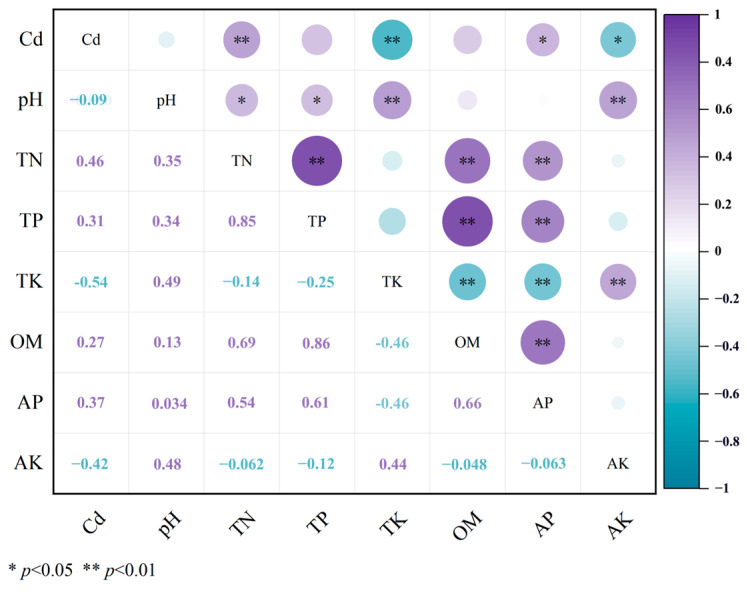
Correlation analysis between plant Cd extraction and soil physicochemical properties.

Plant Cd extraction exhibited a significant positive correlation with TN and AP (Figure 2, *p* < 0.01 and 0.05, respectively). Conversely, significantly negative correlations were observed between plant Cd extraction and TK (*p* < 0.01) as well as AK (*p* < 0.05). Soil pH showed significant positive correlations with TK and AK (*p* < 0.01) as well as TN and TP (*p* < 0.05). TN and TP displayed an extremely significant positive correlation (*p* < 0.01), and both were positively correlated with SOM (*p* < 0.01). Additionally, AP and SOM exhibited a highly significant positive correlation (*p* < 0.01).

## 3. Discussion

### 3.1. Mechanisms of Remediation Efficiency in Woody vs. Herbaceous Plants

Plant growth rate and aboveground biomass serve as critical indicators for assessing phytoremediation capacity [37]. Studies showed that plants exhibited an increasing trend in net growth rate and basal diameter under low Cd concentrations (≤10 mg·kg^−1^), whereas they exhibited a decreasing trend at high Cd levels (≥25 mg·kg^−1^) [38]. Notably, both parameters typically displayed an initial increase followed by a decline with rising Cd concentrations [39]. In this study, both woody and herbaceous plants exhibited growth-promotion trends due to relatively low Cd exposure. Due to their well-developed root systems and high biomass production, woody plants often demonstrate notable Cd tolerance and accumulation capacity, rendering them suitable for long-term Cd immobilization or uptake in phytoremediation applications. In this study, both *P. adenopoda* and *S. babylonica* exhibited a significant increase in shoot biomass with elevated Cd concentrations (*p* < 0.05), while their root biomass remained stable. These findings demonstrate that these woody species exhibit relatively high tolerance to Cd and show potential for the phytoremediation of soils with low Cd contamination, which is consistent with previous studies [40,41]. Additionally, the *BCF* of woody plants exceeded 1 and the *TF* remained below 1, which aligned with the finding that woody plants such as *Melia azedarach*, *Ligustrum lucidum*, and *Viburnum odoratissimum* exhibit a higher *BCF* but lower *TF* [42]. This suggests that woody plants exhibit stronger root accumulation but weaker translocation capacities, likely mediated by Cd immobilization within lignified structures [43,44]. Consequently, despite lower translocation efficiency, woody species can enhance remediation potential through substantial biomass accumulation.

Compared to woody plants, the results of this study indicate that both above- and below-ground biomass of herbaceous plants (*A. hypochondriacus* and *A. argyi*) remained stable under elevated Cd concentrations (*p* > 0.05). This stability may be attributed to a strategy of resource reallocation employed by herbaceous plants under mild Cd stress—prioritizing structural fortification over biomass accumulation [45,46]. This adaptive mechanism enables plants to preferentially allocate limited resources (e.g., photosynthates) towards the development of mechanical support structures (such as basal stem diameter), thereby enhancing resistance to Cd-induced oxidative stress and physical damage [47]. Critically, both species demonstrated a *BCF* and *TF* greater than 1, reflecting superior Cd accumulation and translocation capabilities of those two herbaceous species. These contrasting traits suggest that woody plants are more suitable for in situ Cd immobilization due to their high root *BCF* characteristics, whereas herbaceous species with elevated *TF* values are better suitable for short-rotation phytoextraction via crop rotation, thus forming a dual-mechanism remediation framework. Therefore, future remediation strategies could adopt intercropping systems combining woody and herbaceous plants to synergistically leverage root-mediated immobilization and shoot-based removal mechanisms, thereby enhancing overall remediation efficacy through complementary functional advantages.

### 3.2. Adaptive Mechanisms of Plant Nutrient Uptake Under Cd Contamination

Cd, a non-essential element for plants, induces severe physiological and biochemical disruptions even at trace concentrations [48]. Cd contamination impairs plant nutrient uptake, translocation, partitioning, and metabolic processes [49,50]. Plants would regulate nutrient acquisition strategies, modulating internal elemental stoichiometry to maintain physiological homeostasis under Cd stress [51]. In this study, shoot P content showed a significant negative correlation with root Cd content (*p* < 0.05), which may be attributed to competitive uptake of P and Cd in the rhizosphere. Plants likely enhance P acquisition to mitigate Cd toxicity [52]. Furthermore, internal P nutritional status may indirectly regulate the mobility of Cd in rhizosphere soil by influencing the exudation of organic acids and phenolic compounds. P also immobilizes Cd via adsorption, complexation, precipitation, and crystallization, forming root cell wall-bound P-Cd complexes [53]. Furthermore, N, as a critical factor governing crop yield and quality, exhibits a synergistic interaction with Cd uptake and utilization [54]. For instance, Wang et al. demonstrated that nitrate regulates root architecture via its transporter family (e.g., NRT 2.1), enhancing plant stress tolerance and indirectly modulating Cd absorption efficiency [55]. Additionally, elevated N availability promotes shoot biomass accumulation and amplifies transpirational pull, accelerating Cd translocation from roots to aboveground tissues [56]. This is consistent with the significant positive correlation observed in this study between root N content and shoot Cd concentration (*p* < 0.05). Collectively, plants may adapt to Cd stress through nutrient regulation strategies: (1) reducing toxicity via phosphorus-mediated Cd retention in roots and (2) achieving shoot Cd accumulation through nitrogen metabolism-driven translocation mechanisms.

### 3.3. Soil Drivers of Cd Accumulation in Plants

Soil factors regulate plant Cd uptake and accumulation in plants by modulating Cd bioavailability through solubility, speciation distribution, and adsorption properties. Elevated N levels may enhance microbial mineralization processes, releasing NH_4_^+^ and NO_3_^−^, which subsequently induce proton pump-mediated H^+^ secretion to acidify the rhizosphere and lower soil pH. Reduced pH would mobilize carbonate- or oxide-bound Cd into exchangeable fractions, thereby increasing Cd bioavailability and ultimately enhancing plant Cd extraction capacity [57]. Additionally, nitrogen fertilizer enhanced Cd extraction by *Brassica juncea*, which increased with fertilizer dosage [58]. This supports our finding of a positive correlation (*p* < 0.01) between plant Cd extraction amount and TN. PO_4_^3−^, as a major component of AP, competes with Cd^2+^ for adsorption sites on soil colloid surfaces, including clay minerals, iron oxides, and organic matter [59]. Soil colloids can adsorb heavy metal ions. However, when PO_4_^3−^ concentrations in the soil solution increase, it competes with Cd^2+^ for these adsorption sites, thereby diminishing Cd adsorption capacity [60]. This competitive mechanism makes more Cd^2+^ available for plant uptake, leading to an increase in plant Cd extraction, which may explain the significant positive correlation (*p* < 0.05) between plant Cd extraction and AP in this study. It was also found that potash application may have an antagonistic effect on Cd. Upon addition to the soil, K^+^ increases cation concentrations in the rhizosphere soil solution. During root ion uptake, K^+^ and Cd^2+^ compete for shared transporters. Given the finite number of ion uptake sites on roots, elevated K^+^ concentrations promote preferential K absorption, thereby suppressing Cd uptake and accumulation [61,62]. These findings demonstrate that soil nitrogen and phosphorus may enhance plant Cd extraction by increasing Cd bioavailability. These results collectively indicate that the influence of soil environmental factors on plant Cd uptake involves a complex interplay of multiple interacting mechanisms.

## 4. Materials and Methods

### 4.1. Description of the Experimental Site

This experiment was conducted in the Experimental Base of the Yangtze River Rare Plants Research Institute, China Three Gorges Corporation, situated in Yichang City, Hubei Province, China (31°25′ N, 110°50′ E). The study area belongs to a central subtropical monsoon humid climate characterized by distinct seasonal variations in sunlight, thermal resources, and precipitation. The region has a mean annual temperature of 18.4 °C and receives annual precipitation of 1150 ± 50 mm, with over 75% occurring during the monsoon season.

### 4.2. Experimental Materials

Soil for experimentation was collected from an abandoned coal mine site adjacent to Zhimao Highway in Luojia Village (Zigui County, Yichang City, Hubei Province, China) and surrounding adjacent pristine natural areas after a field investigation in March 2024. Plant debris (roots, stems, and leaves) and rock fragments were manually removed, followed by homogenization of the soil through a 60-mesh sieve. The woody species were planted in 53 cm (diameter) × 43 cm (height) containers and 38 cm × 33 cm containers for herbaceous species.

Two woody species (*P. adenopoda* and *S. babylonica*) and two herbaceous species (*A. hypochondriacus* and *A. argyi*) were selected. Two-year-old seedlings of woody species with uniform stem diameters and vigorous growth were supplied by Kangyuan Greening Nursery (Zigui County, Yichang, China). Seeds of herbaceous species were commercially sourced from Fengpan Horticulture Co., Ltd., in Suqian, China.

### 4.3. Experimental Design

Three treatments were established: undisturbed uncontaminated natural soil (T1), a homogenized mixture (1:1, *w*/*w*) of coal mine-contaminated and natural uncontaminated soils (T2), and abandoned coal mine-contaminated soil (T3), with five replicates per each treatment. Baseline soil physicochemical properties are presented in Table 4. Two-year-old seedlings of woody species were transplanted in late April 2024, while herbaceous species were direct-seeded at high-yield cultivation densities. No fertilizers were applied during the experiment, with soil moisture maintained at 80% field capacity through regulated irrigation. In late October 2024, plant tissues were collected for biomass determination and Cd content analysis, and soil samples were collected for physicochemical analysis.

### 4.4. Measurement Metrics and Methodology

#### 4.4.1. Plant Height and Basal Diameter Net Growth

Plant height was measured using a standard straight ruler, while ground diameter was obtained with a high-precision digital caliper. For each treatment, three permanently marked plants were monitored monthly.

Net growth was calculated as follows:

(1) $$\Delta H=H_{2}-H_{1}$$

(2) $$\Delta D=D_{2}-D_{1}$$

- ΔH: net height increment (cm);
- ΔD: net ground diameter increment (mm);
- H_1_/D_1_: initial measurements (before treatment);
- H_2_/D_2_: final measurements (after treatment period).

#### 4.4.2. Plant Biomass

The test plants were uniformly harvested in October 2024, rinsed thoroughly with deionized water, and air-dried to remove surface moisture. Plants were separated into shoots and roots. Samples were oven-dried at 105 °C for 30 min to deactivate enzymes and then dried at 70 °C to constant mass for biomass determination.

#### 4.4.3. Plant Nutrient Element Content

After homogenization using a grinder, samples were sieved (<1 mm mesh). Nitrogen (N) and phosphorus (P) contents were analyzed according to Chinese agricultural standard NY/T 2017-2011 [63,64].

#### 4.4.4. Soil Properties

Soil pH was measured potentiometrically [65]; soil organic matter (SOM) was determined via the potassium dichromate-concentrated sulfuric acid oxidation method [66]; total nitrogen (TN) was analyzed by the Kjeldahl nitrogen method [67]; total phosphorus (TP) was quantified using the alkali fusion–molybdenum antimony colorimetric method [68]; and total potassium (TK) was measured via flame photometry [69]. Available phosphorus (AP) was extracted with 0.5 mol·L^−1^ sodium bicarbonate and analyzed by the molybdenum antimony colorimetric method [70], while available potassium (AK) was extracted with 1 mol/L ammonium acetate and determined using flame photometry [71].

#### 4.4.5. Plant and Soil Cd Content

Roots were immersed in 0.1 M EDTA solution for 10 min to remove surface-adsorbed Cd^2+^, oven-dried at 105 °C for 30 min, and subsequently dried at 70 °C to constant weight. Dried plant samples were ground and sieved through a 100-mesh sieve. Soil samples were air-dried naturally, impurities were removed, and the samples were sequentially ground through a 2 mm nylon sieve followed by a 100-mesh sieve. Plant samples were predigested with concentrated nitric acid and completely digested via ramped heating (120 °C to 160 °C) with hydrogen peroxide assistance. Soil samples were digested using an HNO_3_: HClO_4_ mixed solution. All digested solutions were diluted to constant volume, filtered, and analyzed for Cd content in both plant and soil matrices by inductively coupled plasma mass spectrometry (ICP-MS; model specified in Methods, Thermo Fisher Scientific, Waltham, MA, USA).

#### 4.4.6. Calculation of Plant Bioconcentration Factor (BCF), Translocation Factor (TF), Cd Extraction Amount, and Extraction Efficiency

The *BCF* of Cd in plants is calculated as follows:

(3) $$BCF=\frac{C_{plant}}{C_{soil}}$$

The *TF* of Cd in plants is calculated as follows:

(4) $$TF=\frac{C_{Shoot}}{C_{Root}}$$

Cd uptake per plant is calculated as follows:

(5) $${Cd}_{plant}=\left(C_{Shoot}\times {DW}_{Shoot}\right)+\left(C_{Root}\times {DW}_{Root}\right)$$

The extraction efficiency of Cd in plants is calculated as follows:

(6) $${Cd}_{extraction\;efficiency}=\frac{{Cd}_{plant}}{C_{soil}}\times 100\%$$

Variable definitions are as follows:

- $C_{plant}$: Cd concentration in homogenized whole-plant tissues (mg·kg^−1^, dry weight);
- $C_{soil}$: total cadmium concentration in rhizosphere soil (mg·kg^−1^);
- $C_{shoot}$,$C_{Root}$: Cd concentrations in shoot (stem + leaves) and root tissues (mg·kg^−1^, dry weight);
- ${DW}_{Shoot}$, ${DW}_{Root}$: oven-dried biomass of shoot and root systems (g).

### 4.5. Statistical Analysis

Data processing and calculations were performed using Microsoft Excel 2016, with relevant indices calculated using formulas. Statistical analyses were conducted with IBM SPSS Statistics 27.0. Differences among treatments in *BCF*, *TF*, Cd uptake, and Cd extraction efficiency were assessed by two-way analysis of variance (ANOVA). Relationships between plant Cd extraction and plant nutrient uptake or soil physicochemical properties were examined using Pearson’s correlation analysis. Figures were generated using OriginPro 2021.

## 5. Conclusions

Through a pot experiment, the present study found that both woody plants (*P. adenopoda* and *S. babylonica*) demonstrated robust Cd tolerance and the capacity for long-term Cd immobilization in root systems, coupled with remediation potential through biomass advantage. In contrast, herbaceous plants (*A. argyi* and *A. hypochondriacus*) exhibited superior Cd accumulation and translocation capabilities. Moreover, high levels of soil total nitrogen and available phosphorus appeared to facilitate plant Cd uptake, while high total potassium levels posed antagonistic effects on Cd absorption. Our results highlight that for the remediation of Cd-contaminated reservoir soils, a woody–herbaceous intercropping system could be implemented to synergistically utilize the long-term immobilization capacity of woody plants and the short-term phytoextraction capability of herbaceous species. This integrated approach can exploit the dual advantages of root Cd stabilization and aerial biomass removal, thereby enhancing remediation efficiency, optimizing soil nutrient management, and ultimately improving plant-mediated Cd remediation effectiveness. To verify the practical applicability and long-term efficacy of this strategy, field monitoring trials will be conducted to evaluate plant remediation performance under natural conditions, thereby enhancing the practical value of this study.

## Figures and Tables

**Table 1:** Effects of Cd on plant growth conditions.

Plant Name	Treatments	Plant Height Net Growth (cm)	Basal Diameter Net Growth (mm)	Aboveground Biomass (g)	Belowground Biomass (g)
*P. adenopoda*	T1	103.33 ± 13.07 a	13.1 ± 1.30 a	53.57 ± 15.51 b	25.92 ± 6.96 a
	T2	105.00 ± 23.28 a	14.8 ± 3.40 a	97.01 ± 16.83 a	43.34 ± 6.46 a
	T3	106.33 ± 5.91 a	16.1 ± 1.50 a	120.85 ± 37.19 a	42.80 ± 13.27 a
*S. babylonica*	T1	104.00 ± 2.16 a	13.2 ± 1.30 a	54.23 ± 4.71 c	33.86 ± 4.04 a
	T2	106.00 ± 10.98 a	15.4 ± 0.40 a	87.82 ± 16.51 b	33.84 ± 6.27 a
	T3	113.67 ± 10.87 a	15.6 ± 2.20 a	96.81 ± 17.18 a	33.38 ± 7.00 a
*A. hypochondriacus*	T1	65.33 ± 12.81 b	5.6 ± 1.30 b	79.43 ± 15.90 a	13.93 ± 5.50 a
	T2	91.33 ± 9.43 ab	7.8 ± 0.80 ab	101.78 ± 36.29 a	13.12 ± 11.20 a
	T3	102.67 ± 6.85 a	8.9 ± 0.60 a	106.10 ± 48.96 a	16.31 ± 3.84 a
*A. argyi*	T1	54.33 ± 7.32 a	4.2 ± 0.30 b	63.04 ± 45.58 a	33.35 ± 21.33 a
	T2	55.67 ± 8.81 a	4.8 ± 0.60 ab	66.23 ± 15.33 a	35.60 ± 3.39 a
	T3	60.00 ± 7.87 a	5.4 ± 1.00 a	73.15 ± 5.36 a	33.89 ± 2.53 a

Note: Different lowercase letters within the same column denote significant differences (*p* < 0.05) in growth parameters for the same species across Cd treatment levels.

**Table 2:** Bioconcentration and translocation factors of Cd by plants.

Plant Name	*BCF*			*TF*		
	T1	T2	T3	T1	T2	T3
*P. adenopoda*	2.28 ± 0.17 AA	1.88 ± 0.23 Ab	1.88 ± 0.19 Ab	0.41 ± 0.09 Ab	0.34 ± 12.13 Ac	0.31 ± 0.03 Ac
*S. babylonica*	0.78 ± 0.15 Bb	2.75 ± 0.48 ABA	6.42 ± 0.90 AA	0.45 ± 0.17 Ab	0.52 ± 0.20 Ac	0.87 ± 0.07 Ac
*A. hypochondriacus*	2.56 ± 0.38 AA	1.65 ± 0.11 Bb	1.39 ± 0.180 Bb	1.72 ± 0.36 BA	1.75 ± 0.20 Bb	3.43 ± 0.45 AA
*A. argyi*	2.57 ± 0.68 AA	3.11 ± 0.09 AA	1.41 ± 0.14 Bb	2.50 ± 0.85 AA	2.31 ± 0.27 AA	1.58 ± 0.28 Ab

Note: Different uppercase letters within the same row indicate statistically significant differences (*p* < 0.05) in Cd *BCF* or *TF* among different Cd treatments for the same plant species. Different lowercase letters within the same column denote significant differences (*p* < 0.05) in *BCF* or *TF* among plant species under the same Cd treatment level. This notation convention applies to subsequent tables unless otherwise stated.

**Table 3:** Cd extraction amount and extraction efficiency in plants.

Plant Name	Cd Uptake (mg·kg^−1^)			Cd Extraction Efficiency (%)		
	T1	T2	T3	T1	T2	T3
*P. adenopoda*	13.62 ± 4.03 CA	36.71 ± 0.23BA	61.11 ± 12.20 Ab	2.28 ± 0.17 AA	1.88 ± 0.23 Ab	1.88 ± 0.19 Ab
*S. babylonica*	6.173 ± 0.998 BA	70.13 ± 11.29BA	224.93 ± 70.13 AA	0.728 ± 0.47 Cb	2.94 ± 0.46 BA	6.42 ± 0.90 AA
*A. hypochondriacus*	12.16 ± 2.60 AA	43.99 ± 13.28 AA	53.46 ± 24.14 Ab	2.56 ± 0.38 AA	1.65 ± 0.11 Bb	1.39 ± 0.18 Bb
*A. argyi*	16.39 ± 7.12 AA	66.26 ± 40.08 AA	25.58 ± 1.01 Ab	2.57 ± 0.68 AA	3.11 ± 0.09 AA	1.41 ± 0.14 Bb

Note: Different uppercase letters within the same row indicate statistically significant differences (*p* < 0.05) in Cd Uptake or Cd Extraction Efficiency among different Cd treatments for the same plant species. Different lowercase letters within the same column denote significant differences (*p* < 0.05) in Cd Uptake or Cd Extraction Efficiency among plant species under the same Cd treatment level. This notation convention applies to subsequent tables unless otherwise stated.

**Table 4:** Basic physical and chemical properties of the test soil.

Treatments	Cd	pH	TN	TP	TK	SOM	AP	AK
	(mg·kg^−1^)		(g·kg^−1^)	(g·kg^−1^)	(g·kg^−1^)	(g·kg^−1^)	(mg·kg^−1^)	(mg·kg^−1^)
T1	0.18 ± 0.01	7.54 ± 0.04	0.32 ± 0.12	0.23 ± 0.01	27.43 ± 0.65	6.98 ± 0.26	6.59 ± 3.96	92.84 ± 8.77
T2	0.35 ± 0.01	7.59 ± 0.01	0.55 ± 0.10	0.61 ± 0.05	25.00 ± 0.80	12.84 ± 0.61	4.53 ± 0.62	85.74 ± 1.97
T3	0.54 ± 0.04	7.58 ± 0.07	1.08 ± 0.06	1.23 ± 0.16	22.41 ± 0.57	20.65 ± 0.28	7.24 ± 0.48	67.31 ± 0.15

## Data Availability

The data presented in this study is available on request from the corresponding author.

## References

[1] Cheng H., Wang X.X., Jiang Y., Chen C.D., Wang Y., Lv M.Q. (2015). Research progress on the effects of the Three Gorges Reservoir on the ecological environment. Chin. J. Eco-Agric..

[2] Zou H. (2025). Study on the Adaptation of Riparian Zone Plants in the Reservoir Area Under the Influence of the Three Gorges Project. Ph.D. Thesis.

[3] Zhou P., Wen A.B., Shi Z.L., Long Y. (2017). Distribution characteristics and pollution evaluation of soil heavy metals of different land use types in Three Gorges Reservoir Region. Trans. Chin. Soc. Agric. Mach..

[4] Shuang Y., Li H., Yang Z.H., Yi Z.W., Li H. (2024). Geochemical characteristics of heavy metal elements in soil-crops in the distribution area of siliceous rocks of the Permian Gufeng Formation in the Three Gorges Reservoir area. Chin. J. Environ. Eng..

[5] Liu Y.J., Li C.X., Mei N., Zhang M.P., Zhang C., Wang D.Y. (2023). Characteristics and risk evaluation of heavy metal contamination in paddy soils in the Three Gorges Reservoir Area. Environ. Sci..

[6] Xu D., Gao B., Gao L., Zhou H., Zhao X., Yin S. (2016). Characteristics of cadmium remobilization in tributary sediments in Three Gorges Reservoir using chemical sequential extraction and DGT technology. Environ. Pollut..

[7] Fang Z., Wang Y., Xie D., Wang D. (2020). Potential ecological risk of heavy metals in a typical tributary of the Three Gorges Reservoir. Bull. Environ. Contam. Toxicol..

[8] Ye C., Li S., Zhang Y., Zhang Q. (2011). Assessing soil heavy metal pollution in the water-level-fluctuation zone of the Three Gorges Reservoir, China. J. Hazard. Mater..

[9] Jiang C.C., Yu G.H., Zhou X.J., Sun F.S., Liu C.-Q. (2024). Biogeochemical process governing cadmium availability in sediments of typical coastal wetlands driven by drying-wetting alternation. J. Hazard. Mater..

[10] Wang C.B., Fang L.Q., Ran C.M., Tang Y.L., Zou Q.H. (2018). Morphological features of Pb and Cd in the water-level fluctuation zone in Fuling section of the Three Gorges Reservoir. Ecol. Environ. Monit. Three Gorges.

[11] Pei S., Jian Z., Guo Q., Ma F., Qin A., Zhao Y., Xin X., Xiao W. (2018). Temporal and spatial variation and risk assessment of soil heavy metal concentrations for water-level-fluctuating zones of the Three Gorges Reservoir. J. Soils Sediments.

[12] Kou T. (2019). Summary of cadmium contaminated site remediation technology. Environ. Prot. Front..

[13] Chen M.N., Nie X.Q., Zhang X.F., He C.Q., Gao B. (2023). Effects of earthworm, straw, and citric acid on the remediation of Zn, Pb, and Cd contaminated soil by *solanum Photeinocarpum* and *Pterocypsela indica*. Environ. Sci..

[14] Latif A., Abbas A., Iqbal J., Azeem M., Asghar W., Ullah R., Bilal M., Arsalan M., Khan M., Latif R. (2023). Remediation of environmental contaminants through phytotechnology. Water. Air. Soil Pollut..

[15] Liu S., Wang C., Yang J., Zhao Q. (2014). Assessing the heavy metal contamination of soils in the water-level fluctuation zone upstream and downstream of the Manwan Dam, Lancang River. J. Soils Sediments.

[16] Mu Y., Zhang C., Li Y., Zhou W., Li Y., Zhao G., Su P. (2024). Research Progress on Physical and Chemical Remediation Methods for the Removal of Cadmium from Soil. Separations.

[17] Almeaiweed N.H., Aloud S.S., Alotaibi K.D., Alotaibi F., Alshebel B. (2025). Enhancing phytoremediation of heavy metal-contaminated aridic soil using olive mill wastewater, sulfur, and chelating agents. Sustainability.

[18] Yu Q., Zhang Z.C., Wang M.Y., Scavo A., Schroeder J.I., Qiu B.S. (2021). Identification and characterization of SaeIF1 from the eukaryotic translation factor SUI1 family in cadmium hyperaccumulator *Sedum alfredii*. Planta.

[19] Sheoran V., Sheoran A.S., Poonia P. (2010). Role of hyperaccumulators in phytoextraction of metals from contaminated mining sites: A review. Crit. Rev. Environ. Sci. Technol..

[20] Khan A.H.A., Kiyani A., Santiago-Herrera M., Ibáñez J., Yousaf S., Iqbal M., Martel-Martín S., Barros R. (2023). Sustainability of phytoremediation: Post-harvest stratagems and economic opportunities for the produced metals contaminated biomass. J. Environ. Manag..

[21] Anggraini Z., Nurliati G., Pratama H.A., Sriwahyuni H., Sumarbagiono R., Shadrina N., Mirawaty M., Pamungkas N.S., Putra Z.P., Yusuf M. (2025). A critical review about phytoremediation of heavy metals and radionuclides: From mechanisms to post-remediation strategies. Chemosphere.

[22] Ogunsola S.S., Oladele O.L., Abdulraheem T., Cooke C.G. (2025). Synergizing phytoremediation and geopolymerization: A sustainable waste-to-wealth approach for heavy metal-contaminated soils. Chemosphere.

[23] Whiting S.N., Leake J.R., McGrath S.P., Baker A.J.M. (2000). Positive responses to Zn and Cd by roots of the Zn and Cd hyperaccumulator Thlaspi caerulescens. New Phytol..

[24] Liu Z., Chen Q., Lin M., Chen M., Zhao C., Lu Q., Meng X. (2022). Electric field-enhanced cadmium accumulation and photosynthesis in a woody ornamental hyperaccumulator—*Lonicera japonica* Thunb. Plants.

[25] Kama R., Ma Q., Nabi F., Aidara M., Huang P., Li Z., He J., Diatta S., Li H. (2023). Hyperaccumulator *Solanum nigrum* L. intercropping reduced rice cadmium uptake under a high-bed and low-ditch planting system. Plants.

[26] Xu L., Tian S., Hu Y., Zhao J., Ge J., Lu L. (2023). Cadmium contributes to heat tolerance of a hyperaccumulator plant species *Sedum alfredii*. J. Hazard. Mater..

[27] Luo J., Yin D., Cheng H., Davison W., Zhang H. (2018). Plant-induced changes to rhizosphere characteristics affecting supply of Cd to Noccaea caerulescens and Ni to *Thlaspi goesingense*. Environ. Sci. Technol..

[28] Zhang X.F., Wu P., Feng J.F., Guo Y.H., Gao B. (2021). Species, habitat characteristics, and screening suggestions of cadmium hyperaccumulators in China. Environ. Sci..

[29] Song B., Zhang Y.X., Tian M.L., Yang Z.J., Wang F.P., Chen T.B. (2019). Potential for cadmium contaminated farmland remediation with *Amaranthus hypochondriacus* L.. Chin. J. Environ. Eng..

[30] Ling T., Jun R., Fangke Y. (2011). Effect of cadmium supply levels to cadmium accumulation by Salix. Int. J. Environ. Sci. Technol..

[31] Liu N., Zhao J., Du J., Hou C., Zhou X., Chen J., Zhang Y. (2024). Non-phytoremediation and phytoremediation technologies of integrated remediation for water and soil heavy metal pollution: A comprehensive review. Sci. Total Environ..

[32] Duan C.X., He P., Wang A.R., Fang W., Xie Y.Z. (2014). Study on tree species selection and allocation mode in the construction of high slope forest land in Zhaomushan Forest Park. J. Southwest China Norm. Univ..

[33] Rebele F., Lehmann C. (2011). Phytoextraction of cadmium and phytostabilisation with Mugwort (*Artemisia vulgaris*). Water. Air. Soil Pollut..

[34] Zhang Z., Zhong J., Guo X., Xu C., Huang D., Liu J., Chen X. (2024). Differential Effects of pH on Cadmium Accumulation in *Artemisia argyi* Growing in Low and Moderately Cadmium-Contaminated Paddy Soils. Chem. Biol. Technol. Agric..

[35] Han M., Ullah H., Yang H., Yu G., You S., Liu J., Chen B., Shahab A., Antoniadis V., Shaheen S.M. (2023). Cadmium uptake and membrane transport in roots of hyperaccumulator *Amaranthus hypochondriacus* L.. Environ. Pollut..

[36] Migeon A., Richaud P., Guinet F., Chalot M., Blaudez D. (2009). Metal accumulation by woody species on contaminated sites in the north of France. Water. Air. Soil Pollut..

[37] Utmazian M.N.D.S., Wenzel W.W. (2007). Cadmium and zinc accumulation in willow and poplar species grown on polluted soils. J. Plant Nutr. Soil Sci..

[38] Luo Z.B., He J., Polle A., Rennenberg H. (2016). Heavy metal accumulation and signal transduction in herbaceous and woody plants: Paving the way for enhancing phytoremediation efficiency. Biotechnol. Adv..

[39] Zhang D.W., Cui J.G., Ge S.F., Yang C.C. (2008). Effect of Cd contamination in soil on growth of poplar of different varieties. Bull. Soil Water Conserv..

[40] Sarfaraz A. (2018). Inter-Species Physiological Response and Phytoremediation Potential of Three Poplar Species Under Cadmium Stress. Ph.D. Thesis.

[41] Kang W., Bao J.G., Zheng J., Zou T., Min J.H., Yang Y.Q. (2014). Analysis on heavy metal enrichment ability of woody plants at ancient copper mine site in Tonglushan of Hubei Province. J. Plant Resour. Environ..

[42] Franke R., Schreiber L. (2007). Suberin—A biopolyester forming apoplastic plant interfaces. Curr. Opin. Plant Biol..

[43] Galvis D.A., Jaimes-Suárez Y.Y., Molina J.R., Ruiz R., Carvalho F.E.L. (2023). Cadmium uptake and allocation in wood species associated to cacao agroforestry systems and its potential role for phytoextraction. Plants.

[44] Moore R.E.T., Ullah I., de Oliveira V.H., Hammond S.J., Strekopytov S., Tibbett M., Dunwell J.M., Rehkämper M. (2020). Cadmium isotope fractionation reveals genetic variation in Cd uptake and translocation by *Theobroma cacao* and role of Natural Resistance-Associated Macrophage Protein 5 and Heavy Metal ATPase-family transporters. Hortic. Res..

[45] Chen C.Z., Lv X.F., Li J.Y., Yi H.Y., Gong J.-M. (2012). Arabidopsis NRT1.5 is another essential component in the regulation of nitrate reallocation and stress tolerance. Plant Physiol..

[46] Luo P., Wu J., Li T.-T., Shi P., Ma Q., Di D.-W. (2024). An overview of the mechanisms through which plants regulate ROS homeostasis under Cadmium stress. Antioxidants.

[47] Yang Y., Zhao Y., Pan M., Yu Y., Guo Y., Ge Q., Hao W. (2024). Physiology and transcriptome analysis of *Artemisia argyi* adaptation and accumulation to soil cadmium. Environ. Saf..

[48] Huang D., Gong X., Liu Y., Zeng G., Lai C., Bashir H., Zhou L., Wang D., Xu P., Cheng M. (2017). Effects of calcium at toxic concentrations of cadmium in plants. Planta.

[49] Thomas M. (2021). A comparative study of the factors affecting uptake and distribution of Cd with Ni in barley. Plant Physiol. Biochem..

[50] Haider F.U., Liqun C., Coulter J.A., Cheema S.A., Wu J., Zhang R., Wenjun M., Farooq M. (2021). Cadmium toxicity in plants: Impacts and remediation strategies. Ecotoxicol. Environ. Saf..

[51] Jia Y., Qin D., Zheng Y., Wang Y. (2023). Finding balance in adversity: Nitrate signaling as the key to plant growth, resilience, and stress response. Int. J. Mol. Sci..

[52] Chtouki M., Naciri R., Soulaimani A., Zeroual Y., El Gharous M., Oukarroum A. (2021). Effect of cadmium and phosphorus interaction on tomato: Chlorophyll a fluorescence, plant growth, and cadmium translocation. Water. Air. Soil Pollut..

[53] Zhu Y.X., Zhuang Y., Sun X.H., Du S.T. (2023). Interactions between cadmium and nutrients and their implications for safe crop production in Cd-contaminated soils. Crit. Rev. Environ. Sci. Technol..

[54] Tian Q., Zheng D., Chen P., Yuan S., Yi Z. (2025). The effects of reducing nitrogen and increasing density in the main crop on yield and cadmium accumulation of ratoon rice. Agronomy.

[55] Ge A.-H., Wang E. (2025). Exploring the plant microbiome: A pathway to climate-smart crops. Cell.

[56] Feng H., Guo J., Peng C., Kneeshaw D., Roberge G., Pan C., Ma X., Zhou D., Wang W. (2023). Nitrogen addition promotes terrestrial plants to allocate more biomass to aboveground organs: A global meta-analysis. Glob. Change Biol..

[57] Lin L., Wu X., Deng X., Lin Z., Liu C., Zhang J., He T., Yi Y., Liu H., Wang Y. (2024). Mechanisms of low cadmium accumulation in crops: A comprehensive overview from rhizosphere soil to edible parts. Environ. Res..

[58] Wang Y.H., Ai S.Y., Li M.J., Yang S.H., Yao J.W., Tang M.D., Zeng Z.B. (2010). Effect of nitrogen fertilization on cadmium translocation in soil-mustard system. Chin. J. Eco-Agric..

[59] Li Z., Huang B., Huang J., Chen G., Zhang C., Nie X., Luo N., Yao H., Ma W., Zeng G. (2015). Influence of Removal of Organic Matter and Iron and Manganese Oxides on Cadmium Adsorption by Red Paddy Soil Aggregates. RSC Adv..

[60] Xia Z., Zhang S., Cao Y., Zhong Q., Wang G., Li T., Xu X. (2019). Remediation of cadmium, lead and zinc in contaminated soil with CETSA and MA/AA. J. Hazard. Mater..

[61] Zamani S., Naderi M.R., Soleymani A., Nasiri B.M. (2020). Sunflower (*Helianthus annuus* L.) biochemical properties and seed components affected by potassium fertilization under drought conditions. Ecotoxicol. Environ. Saf..

[62] Liu Y., Gao J., Zhao Y., Fu Y., Yan B., Wan X., Cheng G., Zhang W. (2024). Effects of different phosphorus and potassium supply on the root architecture, phosphorus and potassium uptake, and utilization efficiency of hydroponic rice. Sci. Rep..

[63] Chen X., Zhang Z., Song X., Deng Z., Xu C., Huang D., Qin X. (2024). Interspecific Root Interaction Enhances Cadmium Accumulation In Oryza Sativa When Intercropping With Cadmium Accumulator *Artemisia argyi*. Ecotoxicol. Environ. Saf..

[64] Wang H.Y., Wang Z.W., Ding R., Hou S.L., Yang G.J., Lü X.T., Han X.G. (2018). The impacts of nitrogen deposition on community N:P stoichiometry do not depend on phosphorus availability in a temperate meadow steppe. Environ. Pollut..

[65] Atilio N.C., Fertonani F.L., Oliveira E.C.d. (2022). Modified and optimized glass electrode for pH measurements in hydrated ethanol fuel. Molecules.

[66] Sergei C., Fu S. (2020). Applications of physical methods in estimation of soil biota and soil organic matter. Soil Ecol. Lett..

[67] Bremner J.M. (1960). Determination of nitrogen in soil by the Kjeldahl Method. J. Agric. Sci..

[68] Sherrell C.G., Saunders W.M.H. (1966). An evaluation of methods for the determination of total phosphorus in soils. N. Z. J. Agric. Res..

[69] Ullah R., Abbas Z., Bilal M., Habib F., Iqbal J., Bashir F., Noor S., Qazi M.A., Niaz A., Baig K.S. (2022). Method development and validation for the determination of potassium (K_2_O) in fertilizer samples by flame photometry technique. J. King Saud Univ.-Sci..

[70] Iatrou M., Papadopoulos A., Papadopoulos F., Dichala O., Psoma P., Bountla A. (2014). Determination of soil available phosphorus using the Olsen and Mehlich 3 methods for greek soils having variable amounts of calcium carbonate. Commun. Soil Sci. Plant Anal..

[71] Hao Z.M. (2000). Determination of soil available potassium. J. Chifeng Univ..

